# The Wrinkles Characterization in GFRP Composites by Infrared Active Thermography

**DOI:** 10.3390/ma16124236

**Published:** 2023-06-07

**Authors:** Adam Stawiarski, Małgorzata Chwał, Marek Barski, Marcin Augustyn

**Affiliations:** Department of Machine Design and Composite Structures, Faculty of Mechanical Engineering, Cracow University of Technology, Al. Jana Pawła II 37, 31-864 Kraków, Poland; malgorzata.chwal@pk.edu.pl (M.C.); marek.barski@pk.edu.pl (M.B.); marcin.augustyn@pk.edu.pl (M.A.)

**Keywords:** wrinkles, GFRP composites, infrared active thermography, turbine blade

## Abstract

An experimental study has been carried out to assess the effectiveness of infrared thermography in wrinkle detection in composite GFRP (Glass Fiber Reinforced Plastic) structures by infrared active thermography. Wrinkles in composite GFRP plates with different weave patterns (twill and satin) have been manufactured with the use of the vacuum bagging method. The different localization of defects in laminates has been taken into account. Transmission and reflection measurement techniques of active thermography have been verified and compared. The section of a turbine blade with a vertical axis of rotation containing post-manufacturing wrinkles has been prepared to verify active thermography measurement techniques in the real structure. In the turbine blade section, the influence of a gelcoat surface on the effectiveness of thermography damage detection has also been taken into account. Straightforward thermal parameters applied in structural health monitoring systems allow an effective damage detection method to be built. The transmission IRT setup allows not only for damage detection and localization in composite structures but also for accurate damage identification. The reflection IRT setup is convenient for damage detection systems coupled with nondestructive testing software. In considered cases, the type of fabric weave has negligible influence on the quality of damage detection results.

## 1. Introduction

The engineering application of composite structures as an alternative to typical metallic materials is often limited because of the great number of possible failure forms. The design process of the composite parts is inseparably connected with the selection and optimization of the manufacturing method. The failure form in composite materials can occur not only as a consequence of the boundary and loading conditions but also during the manufacturing process. The structures exposed to environmental factors are especially prone to durability reduction and different failure forms evolution during normal service life [1,2]. Thus, the quality control of the composite parts is crucial from the safety point of view. In this paper, the wrinkles of composite laminate layers are considered. This type of structural defect often occurs during the manufacturing of composite parts (especially with the single or double surface curvature) with the use of vacuum techniques. The wrinkles also appear due to bending [3] or compression loading conditions [4]. An in-depth review dealing with different types and mechanisms of origin has been presented by Thor et al. [5] and Rym et al. [6]. Xu et al. [7] reported a visible strength reduction caused by out-of-plane wrinkles in curved multidirectional carbon/epoxy laminates under bending loading conditions. The detailed numerical and experimental analysis of the wrinkle’s influence on the damage mechanism and strength of cross-ply composite beams has been continued by Nader et al. [8]. The combined experimental analysis and numerical simulations have been carried out by Hu et al. [9] to investigate the influence of wrinkles on the failure behavior of curved composite laminates. The effect of tensile and compression strength reduction caused by waviness in the automated fiber placement produced composites has been demonstrated by Woigk et al. [10]. Tensile/compression behavior has also been studied by Nartey et al. [11] in the case of laminates containing wrinkles of varying architectures. The influence of the waviness on the mechanical performance of composites has been summarized by Alves et al. [12], and by Boisse et al. [13]. Regardless of the loading condition types, significant stiffness and strength reduction are observed in composite structures with wrinkles. It indicates the importance of the early detection of wrinkles in real composite structures.

Several nondestructive damage detection methods are applied to this failure form in different types of composite structures [14]. The current literature review in the area of nondestructive testing and evaluation techniques of defects in composites has been presented by Chen et al. [15]. Methods focusing on the wrinkle’s failure form have also been developed. The eddy current method has been applied to the visualization and size estimation of wrinkles in multidirectional CFRP (Carbon Fiber Reinforced Plastic) laminates [16,17]. The array ultrasonic inspection with the algorithm of wrinkles identification in laminated composites has been demonstrated by Zhang et al. [18]. The elastic wave propagation method in composite damage detection has also been considered in composite plates and panels [19,20,21].

The wrinkle detection in CFRP structures by the total focusing method (TFM) has been investigated by Ma et al. [22]. A comparative study on the effectiveness of the ultrasonic damage detection method in delamination and fiber waviness detection has been presented by Vieira Goncalves et al. [23]. In line with the current state of the art in the nondestructive inspection of aerospace composite laminates, infrared thermography (IRT), and thermal image processing techniques have been developed [24,25,26,27]. The problem of wrinkles in aerospace composite structures after the manufacturing process has been a challenge for Boeing and the 787 Dreamliner aircraft at the late stage of the development and production process. The emerging defects have been detected in the fuselage skin. Boing developed and patented infrared thermographic methods for wrinkle characterization in composite structures to improve the defect detection process of composite structures [28]. Similar problems with wrinkles in laminates occur in wind turbine components.

The development and trends in nondestructive testing and evaluation for wind turbine composite blades have been presented by Yang et al. [29] and Song et al. [30]. They highlight the most common failure forms sustained in wind turbine blades and nondestructive testing methods with their advantages and limitations. The effect of defects in composite wind turbine blades has also been investigated by Nelson et al. [31]. The influence of wrinkles on stiffness behavior has been analyzed numerically and experimentally by Mendonca et al. [32], Bender et al. [33], and others. Infrared thermography is a promising method for subsurface defect detection whose effectiveness has been proven in aerospace structures. Application of this inspection and monitoring method in the case of turbine blades in operation cause many arising challenges [34]. On the other hand, the dynamic technology development associated with the accuracy, size, and cost availability of infrared cameras connected with the increasing mobility of inspection systems (infrared cameras mounted in smartphones, robots, and drones) caused an increase of interest the scientists and engineers in this technology. However, many factors may disturb the thermographic measurements, and, as a consequence, it leads to the misinterpretation of the thermograms (disturbing environmental factors, inhomogeneities in thermal response caused by reflections or surface dirt). Thus, it is still important to develop thermographic inspection techniques and study the thermal response of different damage types to improve the effectiveness and accuracy of the inspection and monitoring systems and finally increase the safety of wind turbine constructions.

In this paper, the wrinkle characterization in the GFRP structures by infrared thermography has been taken into account. First of all, the two types of glass woven weave—twill and satin have been applied in the vacuum-assisted production of plates with wrinkles to verify the influence of the material type on the results of thermographic inspections. The different subsurface wrinkle localization has been prepared to include the aspects of the defect position to the accuracy of the infrared inspections. The section of the turbine blade with a vertical axis of rotation containing post-manufacturing wrinkles has been prepared to verify the proposed active thermography measurement technique in the real structure. One part of this model has been manufactured with the use of a gel coat finishing surface containing the surface defect to validate the accuracy of the active thermographic measurement techniques in the case of wrinkle detection with disturbing factors. The transmission and reflection thermographic measurement techniques have been verified in the analyzed models and the real wind turbine blade.

## 2. Materials and Methods

**Figure 1:**
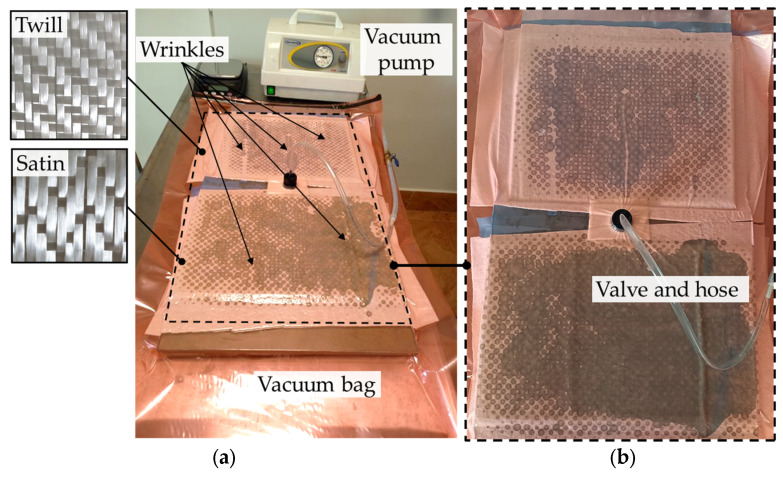
The experimental setup of the flat vacuum bagging technique (**a**) the satin and twill configuration of samples inside the vacuum bag envelope and (**b**) the top view.

**Figure 2:**
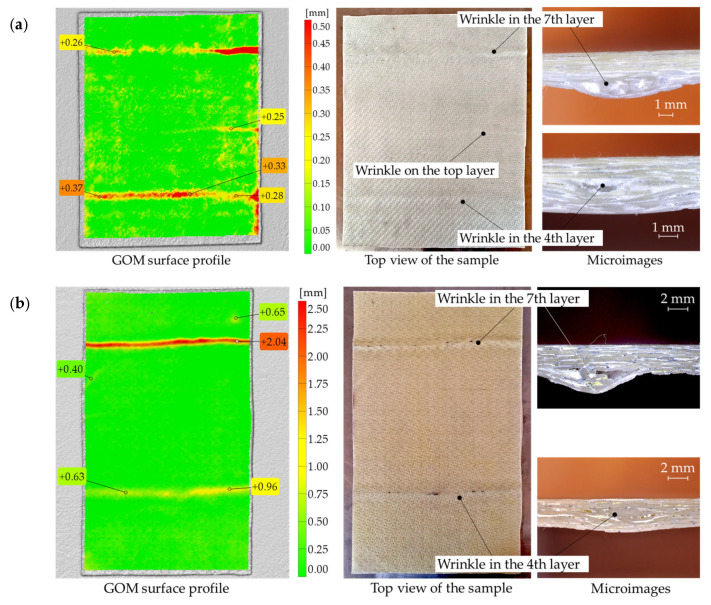
GFRP plates in the case of (**a**) twill, and (**b**) satin layers. From the left: GOM surface profile, top view of the sample, and digital microimages.

Vacuum bagging is a practical method for both large-scale and small-scale applications, such as wind turbine blades, boats, car components, or some hobby projects. This technique has been used to fabricate GFRP samples with wrinkles based on satin and twill fiberglass fabric configurations and epoxy resin. The applied setup of the flat vacuum bagging technique is shown in Figure 1. The two lay-ups were completed at the same time. The GFRP samples consisting of 8 layers have been applied in both cases of fabric configurations. To create the fabrication defects that can be observed after the vacuum bagging manufacturing, the wrinkles have been manually induced in the 4th and 7th layers of the laminate by folding these plies during the hand lay-up process. The induced wrinkles were moved to each other in the plane. During the manufacturing, an additional wrinkle appeared on the top layer (in the middle) of the sample made of twill fabric caused by folding the vacuum bag when the air was pumped out—Figure 1. The obtained GFRP plates of satin and twill configurations having wrinkles are shown in Figure 2. The average thickness of the satin plate was about 3.14 mm, and about 2.54 mm for the twill plate. The fiber volume fraction was assessed by precisely controlling the weight of the particular constituents of a composite structure and finally was equal to 54% for the satin plate and 59% for the twill plate.

The manufactured plates have been scanned with the use of a 3D laser scanner REVscan and measured with the use of GOM Inspect software 2022 SP1. The Creaform REVscan (Amtek Company, Arnold, MD, USA) is a self-positioning, handheld scanner for inspection, quality control, and reverse engineering measurements. The scanner allows to digitize 18,000 points per second with an accuracy of up to 50 µm. The size of the wrinkles measured concerning the composite surface has been demonstrated in Figure 2. The morphology of the analyzed wrinkles has been exposed in the microphotographs added to Figure 2 taken with a CMOS digital microscope (Delta Optical, Warsaw, Poland) supported by the external light source.

**Figure 3:**
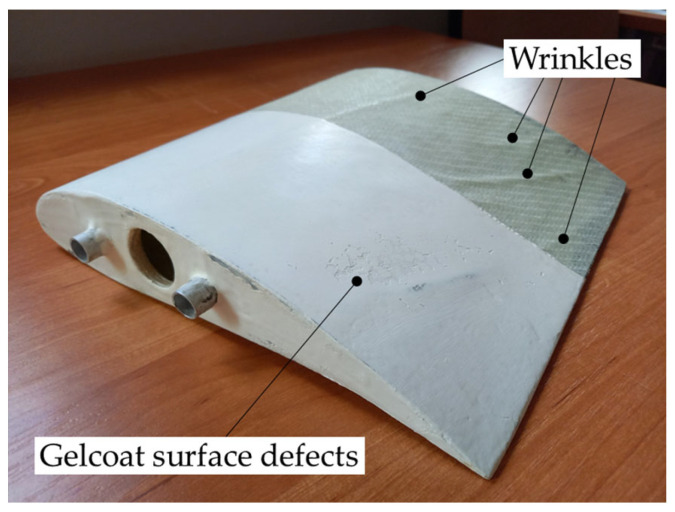
A model of the wind blade with post-manufacturing wrinkles and gelcoat surface defects.

In the next step, vacuum-assisted production was applied to manufacture a section of the real structure of a turbine blade with a vertical axis of rotation containing post-manufacturing wrinkles. The wrinkles appeared during the fabrication of the blade model in the closed mold. The analyzed wind blade model is presented in Figure 3. One part of this model has been manufactured with the use of a polyester gelcoat finishing surface containing the surface defect, which impacts the possibility of wrinkle detection by the active thermographic measurement techniques will also be verified.

Thermography is the process of detection, registration, processing, and visualizing the infrared radiation emitted by non-contact measurements by imaging devices. Active thermography employs an external heat source generating internal heat flow and an increase in temperature to induce relevant thermal contrast between areas of interest.

**Figure 4:**
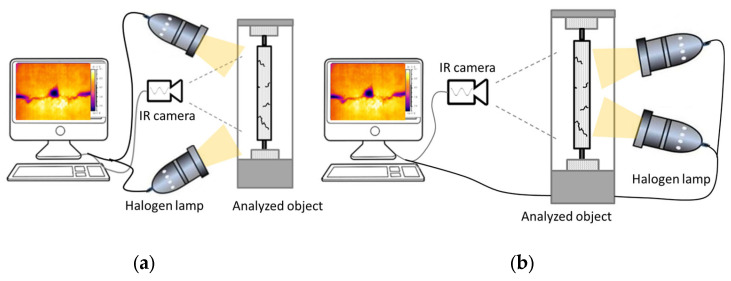
The experimental setup used in the thermographic analyses of composite plates having wrinkles is (**a**) reflection mode and (**b**) transmission mode.

**Figure 5:**
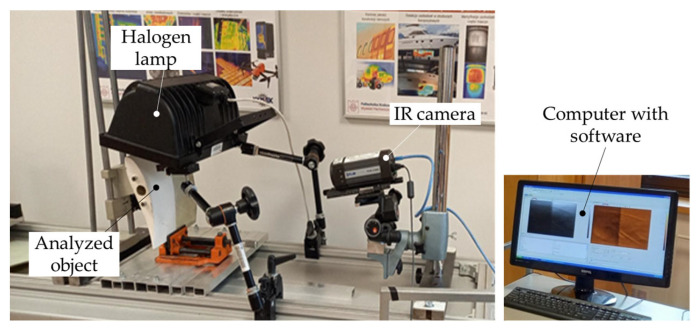
The experimental setup in the reflection mode of the thermographic analysis of the blade model.

The reflection and transmission thermographic methods have been tested to check the possibility, accuracy, and effectiveness of wrinkle detection. The experimental setup schema for the case of two halogen lamps has been demonstrated in Figure 4. Figure 5 shows the experimental setup for the section of the turbine blade wing with the use of one halogen lamp in the reflection technique of measurement. In the thermographic analyses, the FLIR A325 infrared (IR) (FLIR Systems, Wilsonville, OR, USA) camera has been used to record the surface temperature profile. The used IR camera possessed the following parameters: resolution 320 × 240, spectral range 7.5–13 μm, frame rate 60 Hz, and temperature range of −20–350 °C. The data analysis was carried out with the help of IrNDT v1.7.2 software and ThermaCAM Researcher Pro 2.10 software.

The transient thermal analysis has been applied with the use of one 1500 W halogen lamp. The time of the analysis was equal to 300 s, and the time of heating was 30 s. It means that the thermal response of the analyzed structures was monitored both in the heating and cooling process. The measurements were conducted with a frame rate of 9 Hz. The analyzed specimens with wrinkles have been analyzed with the use of one halogen lamp in the transmission and reflection IRT measurement configurations. It should be emphasized that during analyses, manufactured wrinkles were always oriented on the opposite side with respect to the IR camera. It means that convexity was invisible from the observer’s point of view. The IR camera monitored only the flat side of specimens—see microimages in Figure 2. Thus, the worse configuration has been assumed where the defect is invisible from the observer’s point of view.

## 3. Results

### 3.1. The Thermal Analysis of the Composite Plates with Wrinkles

**Figure 6:**
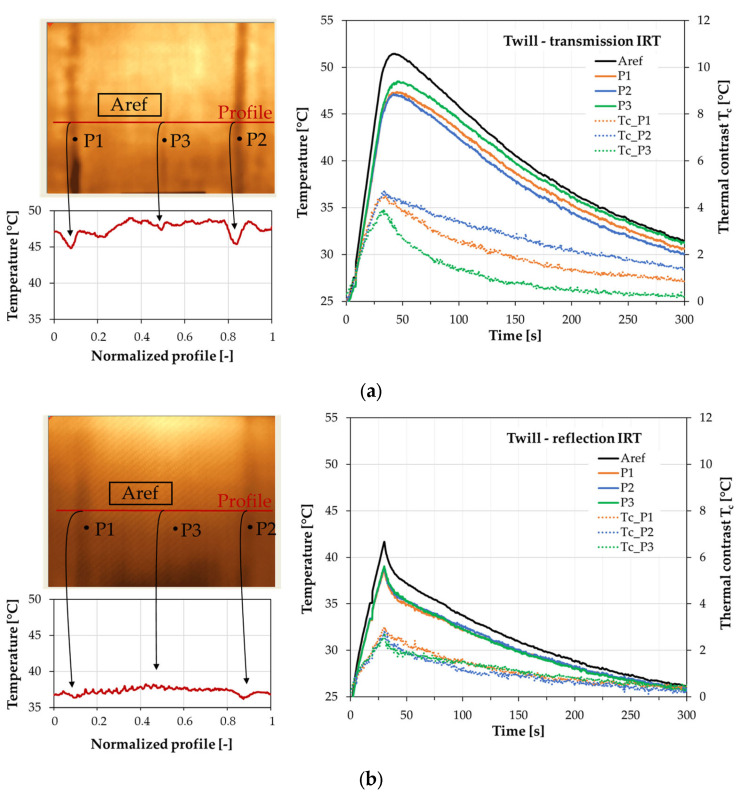
Results of the thermographic analysis of the twill sample (**a**) the transmission mode and (**b**) the reflection mode of the IRT setup. On the left side—the thermal image and temperature distribution of the normalized temperature profile (30 s of analysis). On the right side—temperature and thermal contrast distributions vs. time for defective and reference areas.

The main goal of this work was concerned with the effectiveness of thermography in the case of wrinkle detection in GFRP multilayered composite structures. Firstly, the influence of material type and the depth of the defects in the composite plates on the thermal response has been studied. The two types of glass woven weave, namely twill and satin, having subsurface wrinkles with different localization, have been prepared. In the 8-layer composite plates, the manually induced wrinkles were placed in the fourth and seventh layers. In the case of the twill sample, another wrinkle was made on the top layer caused by folding the vacuum bag when the air was pumped out—Figure 2. Both the satin and twill plates were inspected in two modes of the thermographic setup configurations, namely the transmission and reflection—Figure 4. The transient thermal analysis was assumed for 300 s with a thermal impulse lasting 30 s. The twill sample thermographic results are presented in Figure 6. The thermal image of the twill plate in the case of the transmission IRT has been presented in Figure 6a. In the transmission IRT setup, the temperature profile of the horizontal line placed on the thermal image indicates the position of defects. The temperature profile revealed the wrinkle in the seventh layer (above point P1), in the fourth layer (above point P2), and also the wrinkle on the top layer (above point P3)—Figure 6a. The profile showed the thermal distribution in the 30 s of analysis when the highest thermal contrast was observed. The chart of the temperature profiles in the time domain presents the registered results from points positioned on wrinkles and the average temperature in the reference area (Aref). The possibility of wrinkle detection is visible in the thermal contrast T_c_ distribution versus time. The thermal contrast was computed as the difference between the temperature of the reference area and the temperature of the defective area. In active thermography, the defective areas are detected as areas having a lower temperature. In the transmission setup, the highest thermal contrast of more than 4 °C has been observed for the wrinkle in the fourth layer (point P2). The wrinkle in the seventh layer presented a little lower value of T_c_. The wrinkle on the top layer also revealed the visible difference between the defective and the intact area. The thermographic results of the reflection IRT setup presented in Figure 6b demonstrate much lower thermal contrast T_c_. The wrinkles have been detected; however, only the slight difference between the position of the wrinkles has been revealed. Here, the highest thermal contrast is lower than in the transmission setup. In the reflection mode, the indication of wrinkle position on the temperature profile is not so clear as in the transmission mode. The temperature profile revealed the wrinkle in the seventh layer (above point P1), and in the fourth layer (above point P2), but the wrinkle on the top layer (above point P3) was not so obvious—Figure 6b.

**Figure 7:**
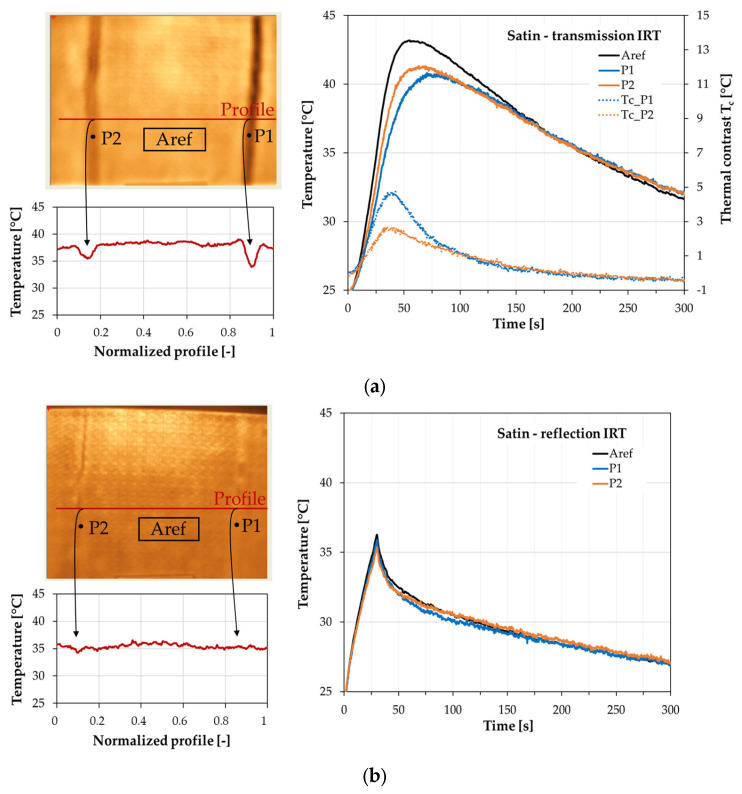
Results of the thermographic analysis of the satin sample (**a**) the transmission mode and (**b**) the reflection mode of the IRT setup. On the left side—the thermal image and temperature distribution of the normalized temperature profile (30 s of analysis). On the right side—temperature and thermal contrast distributions vs. time for defective and reference areas (thermal contrast only for transmission mode).

For satin plate thermograms, point P1 indicates the seventh layer wrinkle, whereas point P2 shows the fourth layer wrinkle—Figure 7. The temperature profile in the transmission setup indicates the location of the wrinkle defects in the satin plate. A sudden change in the surface temperature is observed. The highest thermal contrast is more than 4 °C for the wrinkle in the seventh layer. The deterioration in T_c_ value is observed for the deeper wrinkle in the fourth layer where the maximum value is lower than 3 °C—Figure 7a. The weakening in the possibility of detection of wrinkle defects has been observed for applying the reflection mode of IRT setup—Figure 7b. The disruption of the temperature profile is not so significant to indicate where defects are placed—Figure 7a. The temperature distribution of the reference area and the points positioned on defects are close to each other—Figure 7b.

The comparison between temperature distribution vs. time of the transmission and reflection thermographic setup revealed diverse heating and cooling behavior of plates. The possibility of heating the plates was lower in the case of the reflection mode. In this case, the achieved maximum temperature during the 30 s of heating was about 10 °C lower for the twill plate and about 6 °C lower for the satin plate. Additionally, the heating charts obtained from the applied IRT setups are different. In the transmission mode, after heating, the natural cooling of samples is slow, with smooth characteristics of temperature decay, whereas, in the reflection mode, the temperature after heating decreases quickly in the first part of the cooling, and next, the speed of cooling is much lower.

For the assumed 8-layered plates made of GFRP, the manufactured twill sample possesses lower thickness and higher fiber volume fraction compared with the satin sample.

### 3.2. The Thermal Analysis of the Turbine Blade Section

**Figure 8:**
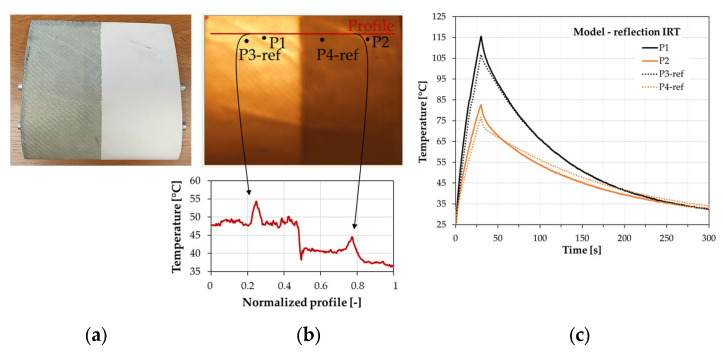
Results of thermographic analysis of a turbine blade section in reflection mode of IRT setup: (**a**) turbine blade, (**b**) thermal image and temperature distributions of normalized temperature profiles, and (**c**) temperature distributions vs. time relevant to defective and reference points.

The section of a turbine blade with a vertical axis of rotation containing post-manufacturing wrinkles has been investigated with the use of both transmission and reflection IRT setup configurations. The measurements were conducted with an IR camera frame rate of 30 Hz setting the heating time at 20 s and the whole time of analysis at 50 s. Figure 8 presents the results of the thermographic inspection with the use of a reflection configuration. The polyester finishing gelcoat surface influences the temperature during the heating process, but the quality of the damage detection process based on the temperature measurement in the time domain and the temperature profile is similar. The difference between temperatures measured at the heating peak at reference points and points localized on wrinkles are similar for both parts of the section.

The temperature profile measured at the gel coat-covered part of the section was lower than for the structure without a finishing surface. However, characteristics of the profile concerning detected wrinkles have been very similar. The heating source localized at the top of the analyzed structure (Figure 5) caused nonuniform temperature distribution on the wind blade section. Nevertheless, the visual verification of the thermographic results allows for identifying the wrinkles for the whole analyzed area. One can also notice that temperatures at points P1 and P2 (points measured on the upper side of wrinkles) are higher than the reference temperatures. The wrinkles have been characterized by convexity facing the heating source. Thus, the upper side of defects has a higher temperature than the lower one. The observed relationship between the temperature points measured for the plates with wrinkles was inverse. For the plated specimens in considered configurations, the temperature in the wrinkled region was lower because the IR camera monitored only the flat side of the samples. Internal defects caused the disturbance in the thermal flows resulting in lower temperatures in thermograms. The localization of the heating source and IR camera and the observed temperature relation between reference and defective areas may also be applied to assess the orientation of the internal defects.

**Figure 9:**
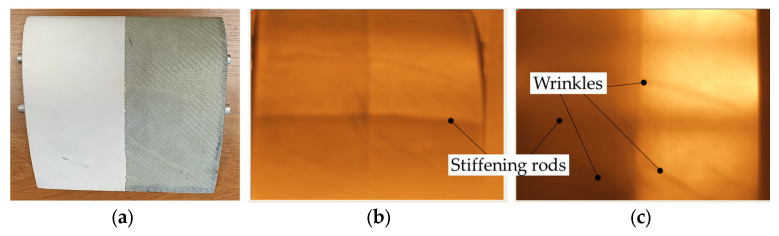
Top view of the section of a turbine blade (**a**) and thermograms obtained from the transmission mode of the IRT setup in the case of (**b**) inner and (**c**) outer position of the halogen lamp.

Figure 9 presents the thermograms of the turbine blade section in the case of the transmission mode of the IRT setup for the inner and outer positions of the halogen lamp. The inner position means that the heating source (halogen lamp bulb) was positioned inside the turbine blade part. The stiffening rods and the wrinkles, both at the covered part of the section and at the part without a finishing surface, were revealed during the thermal analysis.

However, the internal stiffening rods disturbed the wrinkle localization, and the defect analysis was cumbersome. Therefore, the correct analysis should consider the information on the internal structure of the section regardless of the heating source localization.

## 4. Discussion

The results demonstrate the capability of infrared thermography wrinkle detection in different structures made of GFRP composites. Different measurement techniques have been analyzed to verify the effectiveness and accuracy of the damage detection process.

**Figure 10:**
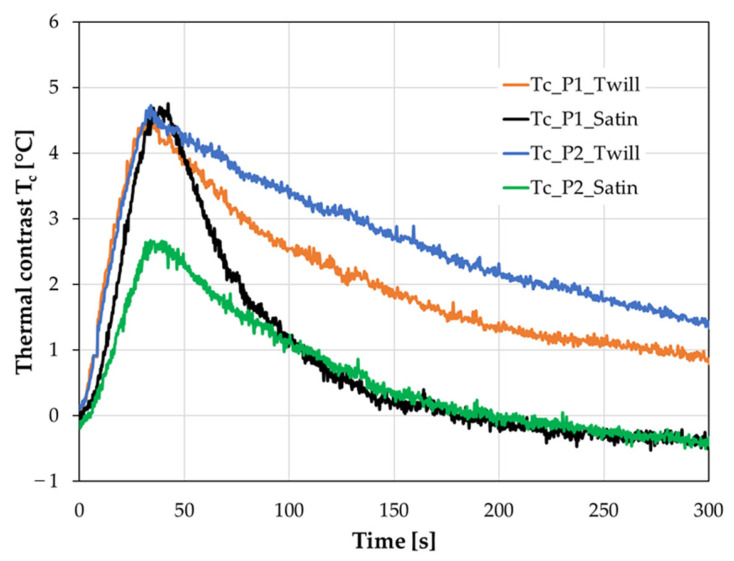
The thermal contrast T_c_ of points located in the seventh layer wrinkle (point P1) and the fourth layer wrinkle (point P2) for twill and satin samples.

Two different glass fiber weaves of the fabric have been analyzed in this research. The influence of the weave type on damage detection results has been negligible. The qualitative assessment of the wrinkle detection based on obtained thermographic results was similar for both materials. From the measurement point of view, it should be noted that the greater thickness of the specimen made of satin fabric results in the lower temperature achieved during the heating process. However, the thermal contrast measured as a difference between temperatures in reference areas and defective points is similar for both glass fabrics—Figure 10.

From the structural health monitoring and inspection point of view, it means that it is not necessary to adapt the methodology of the thermographic inspection to the type of fabric because the effectiveness of the damage detection process is similar.

The methodology of the thermographic inspection determines the effectiveness and quality of the damage detection results. The transmission IRT setup allows us to accurately determine the state of the analyzed structures. The straightforward thermal contrast parameter can be applied here also in the automated damage detection monitoring systems based on the active thermography measurement because the difference between healthy and defective structures is substantial and easy to interpret. The thermal contrast measured in the time domain requires the reference characteristics and may be applied to the mass-produced items’ quality control. The detailed analysis of the intensity of thermal contrast gives accurate information about the position, orientation, and dimension of internal defects. The thermal profile analysis allows satisfying results without the reference structure because each deviation from the temperature level may result from structural defects. Moreover, the detailed and local analysis of the defective areas can be also applied not only in the qualitative damage detection and localization systems but also for quantitative assessment of the damage size, orientation, and localization between laminate layers. The results of the reflection IRT setup depend on the localization and the size of the wrinkle concerning the heat source. The thermographic results of the composite plates with wrinkles demonstrate the problems with the damage assessment in this measurement technique. It should be reminded that the subsurface wrinkles were oriented on the opposite side to the IR camera and the heating source. The measured parameters, namely temperature and thermal contrast in the time domain and the temperature profile, have not allowed for an unambiguous interpretation in the damage detection context. In this case, the inspection of the composite part should be supported by the visual analysis of the temperature distribution during the heating and cooling process and the results presented by the specialized software for thermal image analysis. On the other hand, the analysis of the manufactured section of the wind turbine blade proved that the reflection measurement technique allows for obtaining satisfying results, especially in situations where the transmission configuration is not possible to apply or introduces many complications associated with the geometry of the analyzed structures. The thermographic results of the wing section partially covered by the gelcoat demonstrated lower temperatures achieved in the structures with the additional surface.

In general, the additional layers applied on composites reduce the thermal flow during the active thermography inspection, which may decrease the effectiveness and accuracy of a damage detection system. However, in the analyzed case, the gel coat has not disturbed the quality of the damage detection process. The verification of the covered composite structures requires special attention and testing of the inspection parameters (i.e., heating temperature, time of analysis) because, from a safety and reliability point of view, the crucial issue is to avoid missing substantial defects during thermographic research.

The difference between reference point temperature and temperature on the detected wrinkles was similar in both parts of the section. The temperature profile also indicates a similar temperature disturbance caused by the wrinkle. Moreover, the gelcoat surface defects do not influence the damage detection of internal wrinkles. The damage in the gel coat, which may be identified by visual inspection, does not interfere with the wrinkles in the thermographic inspection, which is especially important from the practical application point of view. The thermographic inspection and quality control of the composite structures revealed internal defects, even if the visual inspection only identified surface damages. It is worth pointing out that the uniform heating process in active thermography is essential in the damage detection process, and it is difficult to achieve in complicated shapes of structures. The transmission IRT setup in the case of the real structure analysis demonstrates the typical problems in the real construction analysis. The internal stiffening elements of the structure may disturb the results and influence misinterpretation in the damage detection and localization context. Regardless of the localization of the heating source (inside or outside the structure), the correct interpretation of the thermographic results requires knowledge about the internal structure of the analyzed part.

## 5. Conclusions

The presented results deal with wrinkle detection in GFRP structures by active thermography inspection in different measurement setup configurations. To summarize obtained results, the following conclusions can be formulated.

- The type of fabric weave has negligible influence on the results of the thermographic inspection. Despite the different thicknesses of the analyzed specimens resulting in different temperatures in the heating process, the thermal contrast indicating the defective areas is similar;
- Straightforward thermal parameters can be applied in structural health monitoring systems. The transmission IRT setup allows for damage detection and localization in composite structures. Detailed analysis of the heating source and IR camera localization and the observed temperature relation between reference and defective areas may also be applied to assess the damage size, orientation, and localization in the laminate layers;
- The reflection IRT setup is convenient for damage detection systems supported by the nondestructive testing software allowing for visual verification of the thermographic results;
- In our case, the gelcoat finishing surface has no substantial influence on the effectiveness of the damage detection process. However, additional coatings reduce the thermal flow during the active thermography inspection; thus, the testing of thermographic parameters is necessary to confirm the accuracy of conducted tests.

**Figure 11:**
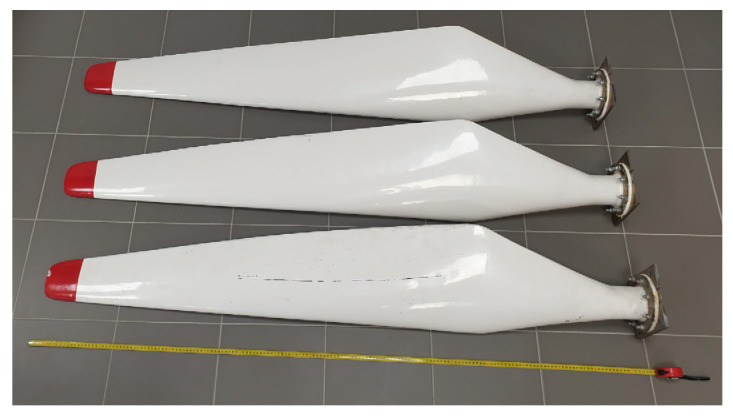
The horizontal axis wind turbine blades (the length equal to 1.75 m).

Further research will focus on the application of active thermography in transmission and reflection configurations in the real blades of wind turbines with the horizontal axis of rotation (Figure 11).

The quality control of horizontal axis wind turbine blades allows for the validation of presented results and the assessment of the active thermography of real constructions.

## Data Availability

Not applicable.
